# Clinical and genetic characteristics of rare congenital adrenal hyperplasia: a retrospective analysis in a Chinese population

**DOI:** 10.3389/fgene.2026.1709951

**Published:** 2026-02-04

**Authors:** Kam Chan, Ying Guo, Shaoling Zhang, Li Yan

**Affiliations:** Department of Endocrinology, Sun Yat-sen Memorial Hospital, Sun Yat-sen University, Guangzhou, China

**Keywords:** congenital adrenal hyperplasia, CYP11A1, CYP11B1, CYP11B2, HSD3B2, StAR

## Abstract

**Objective:**

Rare subtypes of congenital adrenal hyperplasia (CAH) often present with heterogeneous and overlapping clinical features, leading to substantial diagnostic delays and misclassification. This study aimed to characterize the clinical, biochemical, and genetic profiles of rare CAH types in a Chinese cohort and to identify key diagnostic clues that support early differentiation of these uncommon forms.

**Methods:**

We conducted a single-center retrospective study involving 12 confirmed Chinese cases with rare forms of CAH. Clinical data, including phenotypic features, hormonal profiles, and genetic mutations, were meticulously collected and analyzed.

**Results:**

The cohort comprised 11β-hydroxylase deficiency (11-OHD, n = 3), 3β-hydroxysteroid dehydrogenase type 2 deficiency (3β-HSD2D, n = 1), lipoid CAH (LCAH, n = 4), aldosterone synthase deficiency (ASD, n = 2), and 17α-hydroxylase deficiency (17-OHD, n = 2). Distinctive clinical constellations that facilitated subtype differentiation included: low-renin hypertension with hyperandrogenism in 11-OHD; isolated hypospadias without salt-wasting in 3β-HSD2D; life-threatening neonatal salt-wasting with global steroid deficiency in LCAH; salt-wasting without virilization in ASD; and late-onset hypertension with sexual infantilism in 17-OHD. Molecular analysis identified six novel pathogenic variants across the CYP11B1, HSD3B2, StAR, CYP11A1, and CYP11B2 genes, expanding the mutational spectrum.

**Conclusion:**

These results broaden the existing understanding of the mutational landscape underlying rare CAH and reaffirm that comprehensive clinical and genetic evaluation is essential for differentiating these diagnostically challenging subtypes. By improving early detection and enabling more precise, individualized management, this study provides valuable insights that may substantially advance clinical practice and patient care in rare endocrine disorders.

## 1 Introduction

**FIGURE 1:**
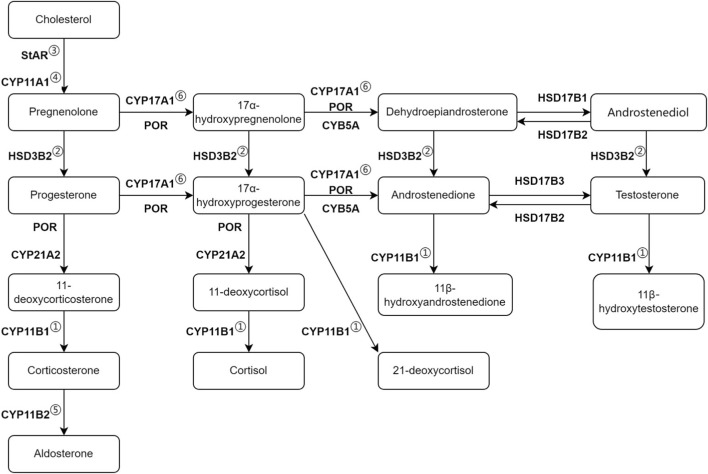
Main steroid synthesis pathways in congenital adrenal hyperplasia. The first and second vertical pathways correspond to the synthesis of mineralocorticoids and glucocorticoids, respectively. The next two columns on the right represent androgen synthesis. ① 11-OHD (Patients P1-P3); ② 3β-HSD2D (Patient P4); ③ LCAH due to StAR mutation (Patients P5-P7); ④ LCAH due to CYP11A1 mutation (Patient P8); ⑤ ASD (Patients P9-P10); ⑥ 17-OHD (Patients P11-P12).

Congenital adrenal hyperplasia (CAH) is an autosomal recessive genetic disorder characterized by impaired synthesis of adrenal corticosteroids due to partial or complete deficiency of a key enzyme involved in steroid hormone synthesis (Auer et al., 2023). The global incidence of classic CAH is estimated to be around 1 in 10,000 to 1 in 20,000 (Speiser et al., 2018; Merke and Auchus, 2020). CAH is classified based on the specific enzyme deficiency involved: 21-hydroxylase deficiency (21-OHD), 11β-hydroxylase deficiency (11-OHD), 17α-hydroxylase/17,20-lyase deficiency (17-OHD), lipoid congenital adrenal hyperplasia (LCAH), P450 oxidoreductase deficiency (PORD), 3β-hydroxysteroid dehydrogenase type II deficiency (3β-HSD2D), and aldosterone synthase deficiency (ASD) (Claahsen-van der Grinten et al., 2022). Among these, 21-OHD is the most common, accounting for over 95% of CAH cases (Speiser et al., 2018). The next most common type is 11-OHD, which accounts for 0.2%–8% of CAH cases, with a relatively higher prevalence among Jews in Morocco and Turkey (Rösler et al., 1992; Güran et al., 2020). The other types, such as 17-OHD, LCAH, PORD, 3β-HSD2, and ASD, are much rarer (Auchus, 2022). The main pathways of steroid production are illustrated in Figure 1. The severity of clinical manifestations in CAH largely depends on the residual enzyme activity. Enzyme deficiencies cause pathway blockages, leading to an imbalance among glucocorticoids, mineralocorticoids, and sex hormones. Reduced cortisol production results in elevated levels of adrenocorticotropic hormone (ACTH), causing bilateral adrenal hyperplasia and hyperpigmentation. The diagnosis of CAH is based on a comprehensive assessment of clinical presentation, biochemical laboratory tests, and genetic analysis (Auer et al., 2023).

Early identification and accurate diagnosis of CAH are crucial for effective clinical management and treatment. With advances in laboratory testing and genetic diagnostic methods, there have been increasing reports on various types of CAH. However, most studies, both domestically and internationally, focus primarily on single types such as 21-OHD, 11-OHD, or 17-OHD, with few comprehensive analyses across types. Therefore, this study collected clinical, biochemical, and genetic data from a Chinese cohort to investigate the rare types of CAH other than 21-OHD. The aim is to enhance understanding of CAH and provide a basis for precise treatment and comprehensive disease management.

## 2 Methods

### 2.1 Patient data

This study included 12 patients with CAH who were hospitalized and treated at the Sun Yat-sen Memorial Hospital, Sun Yat-sen University between 2011 and 2022. The clinical characteristics, laboratory tests, and genetic mutation analysis of these patients were examined. Among them, 10 patients were diagnosed with CAH through genetic testing. Some patients had been previously diagnosed at other hospitals, resulting in some clinical data inconsistencies and incomplete records. This study was approved by the Ethics Committee of Sun Yat-sen Memorial Hospital, Sun Yat-sen University (SYSKY-2024-263-01).

### 2.2 Genetic testing

After obtaining informed consent from the patients and their parents, mutations in the CYP11B1, HSD3B2, StAR, CYP11A1 and CYP11B2 genes were analyzed. DNA was extracted from peripheral blood leukocytes, and the exon regions of CYP11B1, CYP11B2, HSD3B2, StAR, and CYP11A1 were sequenced using Sanger sequencing to identify single-point mutations. For some patients, MLPA was also performed to detect large gene conversions and deletions. All mutations in these five genes were classified according to the Human Genome Variation Society (HGVS) guidelines (https://hgvs-nomenclature.org/) and named using RefSeq sequences: NM_000497.4 (CYP11B1), NM_000198.4 (HSD3B2), NM_000349.3 (StAR), NM_000781.3 (CYP11A1), NM_000498.3 (CYP11B2).

## 3 Results

The clinical characteristics and hormone profiles of all 12 patients at initial diagnosis are summarized in Tables 1, 2. The cohort included 3 patients with 11-OHD (25.0%), 1 patient with 3β-HSD2D (8.3%), 4 patients with LCAH (StAR/CYP11A1 deficiency) (33.3%), 2 patients with ASD (16.7%), and 2 patients with 17-OHD (16.7%).

**TABLE 1:** Clinical characteristics and genotypic of the study patients.

Clinical characteristics	Patient 1(CYP11B1)	Patient 2(CYP11B1)	Patient 3(CYP11B1)	Patient 4(HSD3B2)	Patient 5(StAR)	Patient 6(StAR)	Patient 7(StAR)	Patient 8(CYP11A1)	Patient 9(CYP11B2)	Patient 10(CYP11B2)
Age at diagnosis	3Y	NP	27 Y	10 M	5 Y	5 Y	9 M	8 Y	1 M	1 M
Sex	M	F	F	M	F	F	F	F	M	F
Karyotype	​	46, XX	​	​	46, XX	​	46, XX	​	​	46, XX
Signs and symptoms at diagnosis	Macropenis, rapid growth in height, voice deepening, hirsutism, acne, hyperpigmentation	Ambiguous genitalia	Menstrual abnormalities, hirsutism	Hypospadias	Hyperpigmentation	Hyperpigmentation	Poor weight gain, vomiting, feeding difficulties, hyperpigmentation	Hyperpigmentation, vomiting	Dry skin and mucous membranes	Vomiting, poor responsiveness, developmental delay
Family history	​	​	​	​	The biological sister was born in 2012 and died 2 days after birth	​	The child underwent IVF. The mother has a history of PCOS, and the father has a history of oligospermia. The mother’s brother-in-law has a disorder of sexual development	​	​	​
Initial department of diagnosis	Pediatrics	Gynecology	Endocrinology	Pediatrics	Pediatrics	Pediatrics	Pediatrics	Pediatrics	Pediatrics	Pediatrics
Ht (cm)	122.3 (>+3SD)	112	156.5	73	113	112.5	74.4 (+2.6SD)	128	53.7	67.5 (<-3SD)
Wt (kg)	25 (>+3SD)	21.5	54.6	8.5 (-1SD)	20	17.2	11 (+2SD)	29	3.85	6.5 (<-3SD)
BP(mmHg)	115/76	99/68	129/73	95/57	108/73	99/65	107/71	90/60	99/60	​
External genitalia	Penis 8 × 5 cm, testis 3 mL, PH2	B1PH1	B5PH5	Penis 1.8 × 1.5 cm, PH1	B1PH1	B1PH1	B1PH1	B1PH1	​	B1PH1
ACTH (8a.m., pg/mL)	432	​	22	​	>278.0 pmol/L	76	373	813	15	40
Cortisol (8 a.m., nmol/L)	353.99	​	398.42	​	26.54	1096.46	46.79	40.5	346.88	251.05
17-OHP (ng/mL)	5.61	0.58	2.58	2.73	​	<0.04	<0.03	0.04	1.62	0.41
DHEAS (ug/dL)	55.172	1.072	358.1	1.579	0.01	<0.01	0.41	<4.4	<4.4	​
T (nmol/L)	7.89	<0.35	3.15	<0.087	2.01	<0.0868	<0.087	<0.087	13.28	<0.087
A4 (nmol/L)	>34.9	​	​	​	<1.05	​	0.4537	0.2094	1.2564	​
Ald (ng/L)	59.5	​	81	199	​	17	273	128.7	100.1	60.8
Renin (ng/ml/h)	0.02	​	2.22	16.08	​	0.02	0.08	0	6.57	​
K (mmol/L)	3.89	4.05	4.51	5.28	3.9	3.6	3.58	4.63	6.18	6.18
Na (mmol/L)	140.1	138.2	139.1	137.8	134.7	138.5	141.2	139.6	130.1	130.1
Genotype	Exon1 Exon2-6	c.128G>A (p.Arg43Gln), c.595 + 12G>A	c.441A>T (p.Glu147Asp)	c.1003C>T (p.R335X), c.340G>C (p.A114P)	c.772C>T (p.Gln258Ter), c.784del (p.Gln262ArgfsTer59)	c.714del (p.Lys238AsnfsTer83), c.329G>A (p.S110N)	c.772C>T (p.Gln258Ter)	c.431C>A (p.Ser144Ter)	c.1493C>T (p.THr198Ile), c.1121G>A (p.Arg374Gln)	c.800–2A>G, c.1200 + 1G>A
Homozygous/heterozygous	Homozygous	Heterozygous	Heterozygous	Heterozygous	Heterozygous	Heterozygous	Homozygous	Homozygous	Heterozygous	Heterozygous

F: female; M: male; NP: neonatal period; m: months; y: years; ACTH: adrenocorticotropic hormone; 17-OHP: 17-hydroxyprogesterone; DHEAS: dehydroepiandrosterone sulfate; T: testosterone; A4:androstenedione; Ald: aldosterone; K:potassium; Na: sodium; IVF: *in vitro* fertilization; PCOS: polycystic ovarian syndrome.

**TABLE 2:** Clinical characteristics of 2 Chinese patients with 17-OHD.

Clinical characteristics	Patient 11	Patient 12
Age at diagnosis	19 Y	36 Y
Sex	M	F
Karyotype	46, XY	​
Signs and symptoms at diagnosis	Lack of development of secondary sexual characteristics	Amenorrhea, lack of development of secondary sexual characteristics, male pseudohermaphroditism
Initial department of diagnosis	Endocrinology	Endocrinology
Ht (cm)	158	177
Wt (kg)	53.5	57
BP(mmHg)	140/90	170/110
Tanner	B1PH1	B1PH2
ACTH (8 a.m., pg/mL)	93	149
Cortisol (8 a.m., nmol/L)	23.9	180.84
17-OHP (ng/mL)	0.45	0.41
DHEAS (ug/dL)	<4.4	5.955
T (nmol/L)	1.17	<0.35
A4 (nmol/L)	0.2792	0.4886
Ald (ng/L)	​	876.6
Renin (ng/ml/h)	​	0.42
K (mmol/L)	3	3.07
Na (mmol/L)	145.2	145.3
Adrenal imaging	Bilateral hyperplasia	Bilateral hyperplasia

F: female; M: male; y: years; ACTH: adrenocorticotropic hormone; 17-OHP: 17-hydroxyprogesterone; DHEAS: dehydroepiandrosterone sulfate; T: testosterone; A4:androstenedione; Ald: aldosterone; K:potassium; Na: sodium.

Three patients (P1–P3) were diagnosed with genetically confirmed 11-OHD. All exhibited clinical evidence of androgen excess. Two female patients exhibited signs of virilization, including ambiguous genitalia diagnosed at birth (P2, who underwent vulvoplasty at age 5) and oligomenorrhea with hirsutism in adulthood (P3). Patient P3 had been previously diagnosed with polycystic ovary syndrome (PCOS) on multiple occasions. The male patient (P1) presented with growth acceleration, low-renin hypertension, and signs of peripheral precocious puberty. Biochemically, both patients P1 and P3 demonstrated elevated androgen levels. Genetic testing revealed pathogenic CYP11B1 variants in all cases, including a novel homozygous large deletion involving Exons 1–6 in P1.

One male infant (P4) was diagnosed with 3β-HSD2D following the neonatal identification of hypospadias. Hormone replacement therapy with hydrocortisone was initiated at 1 month of age; consequently, the diagnostic hormone profiles were influenced by treatment. Genetic testing identified compound heterozygous HSD3B2 variants, including a known pathogenic mutation (c.1003C>T) and a novel missense variant (c.340G>C).

Four female patients (P5–P8) were diagnosed with LCAH. All presented with generalized hyperpigmentation. Two infants (P7, P8) displayed severe salt-wasting with vomiting, feeding difficulties, and failure to thrive. Family histories suggestive of adrenal insufficiency or disorders of sex development were noted in two cases (P5, P7). Baseline biochemical evaluation in the untreated patient (P5) demonstrated global steroid deficiency with extremely low cortisol and sex steroid levels and markedly elevated ACTH. Hormonal assessments in patients already receiving glucocorticoid therapy (P6–P8) reflected treatment effects. Genetic defects were found in the StAR gene for three patients (P5-P7), including a novel missense variant (c.329G>A) in P6. One patient (P8) harbored a novel homozygous nonsense mutation in the CYP11A1 gene (c.431C>A).

Two neonates (P9, P10) presented with life-threatening salt-wasting crises characterized by severe hyponatremia, hyperkalemia, dehydration, and feeding difficulties. The male patient exhibited dry skin and mucosal dehydration, while the female patient presented with vomiting, poor responsiveness, and developmental delay. Both were receiving mineralocorticoid replacement at the time of hormone testing. Compound heterozygous CYP11B2 mutations were identified in both patients, including two novel variants (c.1493C>T and c.800–2A>G).

Two patients (P11, P12) were clinically diagnosed with 17-OHD. The 19-year-old male (P11) and 36-year-old female (P12) both exhibited primary sexual infantilism, poorly developed secondary sexual characteristics, and hypertension at presentation. The female patient presented with primary amenorrhea and male pseudohermaphroditism, while the male patient had a female external phenotype with a blind-ending vagina and absent internal genitalia. Both patients had hypokalemia, accompanied by markedly decreased sex hormone levels and low-normal cortisol on laboratory assessment. Imaging studies further identified bilateral adrenal hyperplasia in each patient. Genetic testing was not available for confirmation, and the diagnoses were based on the classic clinical and biochemical phenotype.

## 4 Discussion

This study highlights the considerable diagnostic complexity of rare CAH subtypes, which often present with overlapping manifestations and atypical biochemical patterns. By integrating clinical, hormonal, and genetic evidence, along with the identification of several novel variants, this work contributes meaningful insight into improving early diagnostic accuracy and personalized management strategies.

The diagnostic challenge of 11-OHD, underscored by its frequent mimicry of 21-OHD, was evident in our cohort where all three patients presented with hyperandrogenism. The pivotal discriminating feature was the presence of low-renin hypertension in Patient 1. Previous studies have shown that approximately two-thirds of 11-OHD patients exhibit hypertension (Al-Jurayyan, 1995; Zhou et al., 2020; Sun et al., 2023). This distinct clinical presentation stems from a key pathophysiological divergence: the accumulation of 11-deoxycorticosterone (DOC). In 11-OHD, elevated DOC acts as a potent mineralocorticoid, suppressing the renin-angiotensin system and leading to sodium retention and hypertension, while its concomitant glucocorticoid activity may mask symptoms of cortisol deficiency. This biochemical profile explains the characteristic clinical picture of hypertension concurrent with hyperandrogenism, typically without profound salt-wasting, which is a pattern that helps distinguish 11-OHD from classic salt-wasting 21-OHD. All three patients with 11-OHD in our cohort presented with clinical hyperandrogenism. These patients are often misdiagnosed with conditions such as hirsutism, polycystic ovary syndrome (PCOS), or 21-OHD (Reisch et al., 2013; Bulsari and Falhammar, 2017). These cases reinforce that hypertension in the presence of androgen excess should raise suspicion for 11-OHD. Genetically, our cohort contributes to both the delineation and expansion of the CYP11B1 mutational spectrum. The novel large homozygous deletion encompassing exons 1–6 in Patient 1 represents a severe null allele, consistent with his early-onset, classic presentation featuring both hyperandrogenism and hypertension. In contrast, Patients 2 and 3 carried compound heterozygous mutations (c.128G>A/c.595 + 12G>A and c.441A>T, respectively). The c.128G>A allele has been associated with a non-classical, latent phenotype in other populations (Kim et al., 2013), which may correlate with the prenatal diagnosis and later surgical intervention in Patient 2. The c.441A>T mutation in patient 3 has been previously reported and confirmed as pathogenic (Fisher et al., 2000), combined with a presumably milder second allele, likely underpins her later presentation and initial misdiagnosis as PCOS. These findings align with the broader observation that 11-OHD exhibits a spectrum of severity influenced by genotype, where biallelic severe mutations often lead to classic childhood forms, while the presence of at least one milder allele can attenuate the phenotype (Yildiz et al., 2021). With over 200 mutations reported, primarily missense or nonsense changes in exons 2, 6, 7, and 8 (Sun et al., 2023), establishing precise genotype-phenotype correlations remains challenging but crucial. Our identification of a novel deletion in the Chinese population directly addresses this ongoing effort (Güran et al., 2020; Yildiz et al., 2021; Sun et al., 2023; Liu et al., 2024), emphasizing the need for population-specific data to refine diagnostic strategies and genetic counseling.

3β-HSD2D demonstrates considerable phenotypic heterogeneity (Al Alawi et al., 2019). This was exemplified by Patient 4 in our cohort, a male infant whose sole presenting feature was isolated hypospadias, in contrast to the classic neonatal salt-wasting crisis. The absence of overt adrenal insufficiency led to initial diagnostic delay, highlighting a key challenge in recognizing the non–salt-wasting form of the disorder. The pathophysiology of this attenuated phenotype can be explained by partial preservation of enzymatic activity. In males, reduced activity of type II 3β-HSD in the adrenal glands and testes decreases the production of testosterone and dihydrotestosterone, resulting in varying degrees of genital maldevelopment, such as ambiguous genitalia, micropenis, and hypospadias at birth. Females may experience mild to moderate masculinization due to the conversion of excessive dehydroepiandrosterone to more potent androgens, presenting as clitoral enlargement and early development of pubic hair (Zhang et al., 2000). Crucially, the severity of concomitant mineralocorticoid deficiency varies with the degree of residual enzyme function. Patient 4’s lack of electrolyte disturbances pointed to retained, albeit suboptimal, aldosterone production. Molecular analysis therefore plays a pivotal role in resolving diagnostic ambiguity, especially when biochemical markers are non-specific or unavailable (Nicola et al., 2022). In Patient 4, it revealed compound heterozygous variants: the known c.1003C>T allele, which retains approximately 2% residual activity *in vitro* (Baquedano et al., 2018), and the novel c.340G>C (p.Ala114Pro) variant. This genotype is consistent with a non–salt-wasting phenotype, as mutations preserving >5% residual activity may prevent severe salt-wasting crises (Guran et al., 2020). Our findings suggest the novel c.340G>C variant likely contributes to this milder presentation by encoding a protein with partial function. This case underscores a critical clinical implication: isolated male undervirilization, particularly hypospadias, should prompt consideration of 3β-HSD2D even in the absence of electrolyte abnormalities. Given the diagnostic overlap with other disorders, early genetic testing for HSD3B2 mutations is paramount. It enables a definitive diagnosis, guides appropriate glucocorticoid replacement, and provides essential information for family counseling. The identification of the novel c.340G>C variant expands the mutational spectrum and highlights the need for functional studies to validate its role in partial enzyme deficiency.

The four patients with LCAH in our cohort (P5-P8) demonstrated the phenotypic spectrum of this disorder, ranging from life-threatening neonatal crisis to later-onset chronic presentation. Clinically, two infants (P7, P8) presented with classic salt-wasting crises, while two children (P5, P6) were diagnosed following the investigation of hyperpigmentation and a positive family history, without acute metabolic decompensation. This spectrum, from severe neonatal to later-onset forms, aligns with previous reports and suggests that mutations with residual function may permit delayed clinical presentation (Joshi et al., 2014; Zhao et al., 2018). Biochemically, the diagnosis hinges on the characteristic pattern of global steroid deficiency. Our untreated patient (P5) exhibited this definitive profile: profoundly low or undetectable levels of cortisol, aldosterone, and all sex steroids, accompanied by markedly elevated ACTH, as well as bilateral adrenal enlargement on imaging, further support the diagnosis. This “pan-hyposteroideism” results from a complete blockade at the start of the steroidogenic pathway, distinguishing LCAH from other CAH forms characterized by the accumulation of specific precursors (Bose et al., 1996; Miller, 2017). For patients already on replacement therapy (P6-P8), diagnosis relied on integrating this characteristic clinical history with genetic confirmation. LCAH is primarily caused by mutations in the StAR gene (King et al., 2011), and occasionally by mutations in the CYP11A1 gene, which encodes the P450 cholesterol side chain cleavage enzyme (P450scc) (Hauffa and Hiort, 2011). Genetically, our findings both confirm established patterns and contribute novel data. The p.Gln258Ter variant in StAR was the most frequent in our cohort, consistent with its reported predominance in East Asian populations (Nakae et al., 1997; Kang et al., 2017; Zhang et al., 2021). Importantly, we expanded the mutational spectrum by identifying two novel pathogenic variants: a StAR missense mutation (c.329G>A, p.Ser110Asn) and a homozygous CYP11A1 nonsense mutation (c.431C>A, p.Ser144Ter) in Patient 8. This latter case confirms that defects in the initial step of steroidogenesis can produce a severe neonatal phenotype indistinguishable from classic StAR-deficient LCAH. From a diagnostic standpoint, StAR sequencing should be prioritized, followed by sequencing P450scc analysis when initial results are negative, as necessitated by our Patient P8 (Miller, 2017). Early recognition of this pattern is clinically critical, as prompt initiation of glucocorticoid and mineralocorticoid replacement is life-saving.

The two patients with aldosterone synthase deficiency (ASD) in our cohort (P9 and P10) presented a defining and diagnostically valuable phenotype: severe, isolated mineralocorticoid deficiency. Both presented in infancy with life-threatening salt-wasting crises, fulfilling the classic triad of hyponatremia, hyperkalemia, and elevated plasma renin activity with low aldosterone levels (Miller and Auchus, 2011). Critically, both patients had preserved glucocorticoid and sex steroid production. This clear biochemical dissociation provides the key to distinguishing ASD from other forms of CAH, such as 21-OHD, where mineralocorticoid deficiency is typically accompanied by androgen excess. Molecular analysis confirmed the specificity of the defect and revealed novel genetic causes. Patient 9 carried a novel c.1493C>T variant alongside the known c.1121G>A (p.Arg374Gln) mutation, which has been shown to disrupt hydrogen-bond formation and impair enzyme activity (Nguyen et al., 2010). Patient 10 harbored two novel splice-site variants (c.800–2A>G and c.1200 + 1G>A), both predicted to cause a complete loss of function through aberrant RNA splicing (Li et al., 2016). These variants specifically disrupt the terminal reaction in aldosterone production mediated by CYP11B2, thereby offering a clear genetic rationale for the distinctive hormonal pattern observed, in which the deficit is confined to aldosterone synthesis without affecting other steroid pathways. Therefore, our findings reinforce that an infant with a salt-wasting crisis must undergo a comprehensive hormonal evaluation. The absence of cortisol deficiency and androgen excess should immediately elevate ASD in the differential diagnosis. In this specific clinical context, genetic testing for CYP11B2 transitions from a confirmatory tool to an essential diagnostic procedure, enabling a definitive diagnosis that directs targeted fludrocortisone replacement. The identification of two novel variants in our cohort expands the mutational spectrum of ASD and underscores the utility of molecular analysis in achieving precision diagnosis and management for this distinct CAH subtype.

The two patients with clinically diagnosed 17-OHD in our cohort (P11 and P12) exemplify the diagnostic delays characteristic of this condition, as both were not recognized until adulthood. Their late presentation is consistent with epidemiological data showing that most cases are diagnosed during adolescence or later, often during evaluation for hypertension or disorders of sexual maturation (Fontenele et al., 2018; Kurnaz et al., 2020; Hinz et al., 2018). The diagnosis relies on recognizing a distinctive clinical triad: low-renin hypertension, hypokalemia, sexual immaturity in 46, XX females, and incomplete virilization or pseudohermaphroditism in 46, XY males (Maheshwari et al., 2022). In our patients, this manifested as hypertension with hypokalemia accompanied by complete absence of pubertal development. This constellation reflects the dual enzymatic deficiency of CYP17A1, which blocks cortisol and sex steroid synthesis and leads to compensatory overproduction of mineralocorticoid precursors, resulting in hypertension and renin suppression. Because classic symptoms of glucocorticoid deficiency are often absent, the diagnosis may be overlooked in early childhood. This delay can result in prolonged, untreated hypertension and an increased risk of hypertension-mediated end-organ damage. Accordingly, adolescents or young adults presenting with hypertension and delayed or absent puberty should be evaluated for 17-OHD, with particular attention to serum potassium and renin activity, as these are key diagnostic clues (Fontenele et al., 2018; Kurnaz et al., 2020). In the absence of available genetic testing, the diagnosis in our patients was reliably established through consistent clinical features and a characteristic steroid hormone profile, including elevated mineralocorticoid precursors with low cortisol and sex steroids. This underscores that rigorous clinical and biochemical correlation can enable timely diagnosis and initiation of targeted therapy to control blood pressure and address hypogonadism, even before molecular confirmation.

The primary limitation of this study is its retrospective single-center design, which led to incomplete documentation for some patients at the time of initial presentation. The relatively small sample size also restricts the generalizability of our findings, and the absence of patients with PORD as well as the lack of longitudinal treatment outcome data limit our ability to compare disease trajectories across CAH subtypes. Future research should incorporate larger, multi-center cohorts and prospective data collection to validate these observations and further elucidate the diagnostic and therapeutic implications of rare CAH variants.

## 5 Conclusion

In conclusion, this 12-year single-center analysis provides one of the most detailed clinical characterizations of rare CAH subtypes in China and offers important guidance for their differentiation in routine practice. By systematically summarizing these distinguishing patterns, the study offers practical diagnostic cues that can help clinicians differentiate rare CAH types earlier in the disease course. The identification of six novel pathogenic variants further enriches the mutation spectrum and strengthens genotype–phenotype interpretation for rare CAH forms. Overall, the findings underscore that careful attention to characteristic but frequently overlooked clinical and hormonal patterns is critical for early suspicion and differentiation of rare CAH subtypes. When presentations are atypical or overlapping, comprehensive genetic testing remains indispensable for diagnostic confirmation and for guiding individualized management.

## Data Availability

The original contributions presented in the study are included in the article; further inquiries can be directed to the corresponding author.
